# DNA Damage Inducer Mitoxantrone Amplifies Synergistic Mild‐Photothermal Chemotherapy for TNBC via Decreasing Heat Shock Protein 70 Expression

**DOI:** 10.1002/advs.202206707

**Published:** 2023-04-17

**Authors:** Zuqin Chen, Sunfan Li, Fangzhou Li, Cheng Qin, Xianlei Li, Guangchao Qing, Jinjin Wang, Bozhang Xia, Fuxue Zhang, Liangliang Meng, Xing‐Jie Liang, Yueyong Xiao

**Affiliations:** ^1^ Medical School of Chinese PLA No. 28 Fuxing Road Beijing 100853 P. R. China; ^2^ Department of Radiology The First Medical Center Chinese PLA General Hospital Beijing 100853 P. R. China; ^3^ School of Microelectronics Shanghai University Shanghai 201800 P. R. China; ^4^ CAS Key Laboratory for Biomedical Effects of Nanomaterials and Nanosafety CAS Center for Excellence in Nanoscience National Center for Nanoscience and Technology of China Beijing 100190 P. R. China; ^5^ Department of Medical Imaging Chinese PAP Force Hospital of Beijing Beijing 100600 P. R. China

**Keywords:** chemotherapy, DNA damage, heat shock protein, mild‐photothermal therapy, mitoxantrone, polymeric micelle, triple‐negative breast cancer (TNBC)

## Abstract

Patients with triple‐negative breast cancer (TNBC) have the worst clinical outcomes when compared to other subtypes of breast cancer. Nanotechnology‐assisted photothermal therapy (PTT) opens new opportunities for precise cancer treatment. However, thermoresistance caused by PTT, as well as uncertainty in the physiological metabolism of existing phototherapeutic nanoformulations, severely limit their clinical applications. Herein, based on the clinically chemotherapeutic drug mitoxantrone (MTO), a multifunctional nanoplatform (MTO‐micelles) is developed to realize mutually synergistic mild‐photothermal chemotherapy. MTO with excellent near‐infrared absorption (≈669 nm) can function not only as a chemotherapeutic agent but also as a photothermal transduction agent with elevated photothermal conversion efficacy (*ƞ* = 54.62%). MTO‐micelles can accumulate at the tumor site through the enhanced permeability and retention effect. Following local near‐infrared irradiation, mild hyperthermia (<50 °C) assists MTO in binding tumor cell DNA, resulting in chemotherapeutic sensitization. In addition, downregulation of heat shock protein 70 (HSP70) expression due to enhanced DNA damage can in turn weaken tumor thermoresistance, boosting the efficacy of mild PTT. Both in vitro and in vivo studies indicate that MTO‐micelles possess excellent synergetic tumor inhibition effects. Therefore, the mild‐photothermal chemotherapy strategy based on MTO‐micelles has a promising prospect in the clinical transformation of TNBC treatment.

## Graphical abstract

A multifunctional drug delivery system (MTO‐micelles) based on the chemotherapeutic drug mitoxantrone (MTO) and amphiphilic polymer DSPE‐PEG_2000_ is developed. MTO induced DNA damage amplify the effect of Mild‐photothermal Chemotherapy by downregulating HSP70 expression. This mild‐photothermal chemotherapeutic formulation provides a facile and effective way for the TNBC treatment.

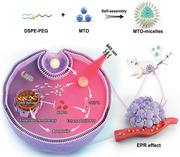

## 1 Introduction

Breast cancer is the most common cancer diagnosed in 2022, according to the American Cancer Society, and it remains the second‐leading cause of cancer‐related death after lung cancer.^[^ ^1^, ^2^ ^]^ Triple‐negative breast cancer (TNBC) refers to nearly 20% of breast cancers with negative expression of estrogen receptor (ER), progesterone receptor (PR), and human epidermal growth factor receptor 2 (HER2), all of which are molecular targets of therapeutic agents.^[^ ^3^ ^]^ Chemotherapy is the primary established systemic treatment for patients with TNBC in both early and advanced stages of the disease. However, patients with TNBC usually have poor clinical outcomes owing to the poor pharmacokinetics and insufficient tumoral delivery of chemotherapeutic agents, and lack of recognized molecules for targeted therapy.^[^ ^4^, ^5^, ^6^ ^]^ Therefore, there is an urgent need to develop better therapeutic strategies for TNBC.

Photothermal therapy (PTT) is a promising therapeutic modality with remarkable therapeutic benefits, including non‐invasiveness, high spatial and temporal selectivity, and temperature controllability.^[^ ^7^ ^]^ Generally, PTT destroys tumors through hyperthermia generated from photothermal transduction agents (PTAs) after local near‐infrared (NIR) light irradiation. To completely ablate tumor cells, temperatures above 50 °C that induce tumor necrosis are necessary, but such a high temperature would also damage the normal tissues around the tumor.^[^ ^8^, ^9^, ^10^, ^11^ ^]^ However, a mild temperature increase (42–49 °C) would prevent apoptosis due to the thermoresistance of tumor cells via upregulation of heat shock proteins (HSPs) expression.^[^ ^12^, ^13^, ^14^ ^]^ Indeed, PTT is not an exception to the rule that a single treatment is insufficient to elicit a robust anticancer response. To address these issues, nanosystems combined with photothermal therapy and chemotherapy have been extensively researched and found to have good mutually synergistic effects.^[^ ^15^, ^16^ ^]^ However, although current PTAs (such as gold nanoparticles, carbon‐based agents, and organic small molecules) have excellent photothermal conversion efficiency,^[^ ^17^ ^]^ they are challenging to fulfill clinical application criteria. Metal nanoparticles, for example, are easy to deposit in vivo, while the metabolism and potential toxicity of organic small molecules are still unknown.^[^ ^18^, ^19^ ^]^ As a result, it is critical to find chemotherapeutic agents with photothermal conversion properties to construct nanoplatforms for combined mild‐photothermal and chemotherapy of tumors.

Mitoxantrone (MTO), a topoisomerase II inhibitor, could even bind with DNA and prevent replication, leading to double strand breaks (DSBs) and interfering with RNA synthesis.^[^ ^20^ ^]^ MTO has been commonly applied as a chemotherapeutic agent in the treatment of breast and prostate cancer, acute leukemias, and lymphomas.^[^ ^21^ ^]^ Besides, studies show that it has strong NIR absorption at 600–700 nm, implying that it has the potential to function as a PTA.^[^ ^22^, ^23^ ^]^ Nevertheless, MTO has some drawbacks, including poor water‐solubility and a lack of tumor targeting, which limits its clinical application.^[^ ^24^, ^25^ ^]^ Nanotechnology is utilized in pharmaceutics to enhance the delivery of therapeutics with low water solubility, high systemic toxicity, and instability.^[^ ^26^, ^27^ ^]^ Polymeric micelles (PMs) have been identified as one of the most promising nano‐drug delivery systems for clinical transformation.^[^ ^28^ ^]^ A variety of chemotherapeutic drug‐loaded PMs have either entered clinical application or undergoing clinical investigation.^[^ ^29^, ^30^, ^31^ ^]^ Thus, integrating chemotherapeutic agents into polymeric nanomicellar systems and taking advantage of the properties of the prolonged systemic circulation and enhanced permeability and retention (EPR) effects of nanoparticles may be a promising approach to overcome their limitations and improve therapeutic efficacy.

**Scheme 1:**
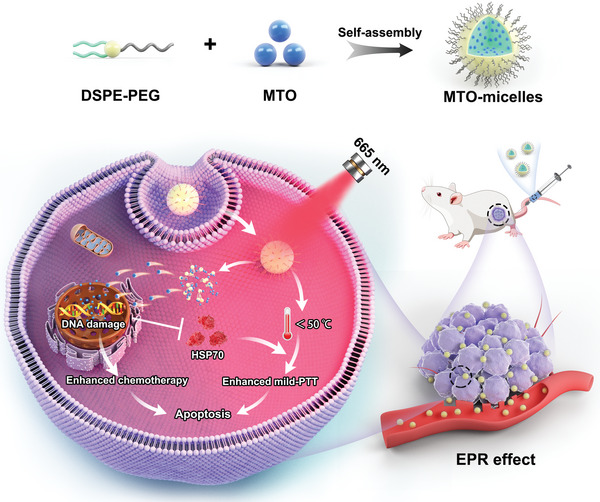
Schematic illustration of the multifunctional MTO‐micelles for mutually synergistic mild‐photothermal chemotherapy of TNBC.

Here, we developed multifunctional nanomicelles (MTO‐micelles) based on MTO and N‐(carbonyl‐methoxy polyethylene glycol 2000)‐1,2‐distearoyl‐sn‐glycerol‐3‐phosphoethanolamine (DSPE‐PEG_2000_) for mutually synergistic mild‐photothermal chemotherapy of TNBC (**Scheme** **1**). After intravenous administration, MTO‐micelles accumulated in the tumor sites via the EPR effect. Following local NIR irradiation, the light energy absorbed by MTO‐micelles was converted into heat for mild‐PTT. The increasing temperature assisted MTO in binding with tumor cell DNA, resulting in chemotherapeutic sensitization. Additionally, the downregulation of HSP70 expression caused by enhanced DNA damage could in turn weaken tumor cell thermoresistance, boosting the efficacy of mild PTT. Therefore, the mild‐photothermal chemotherapy strategy based on MTO‐micelles has a great clinical prospect in the treatment of TNBC.

## 2 Results and Discussion

### 2.1 Characterizations of MTO‐Micelles

**Figure 1:**
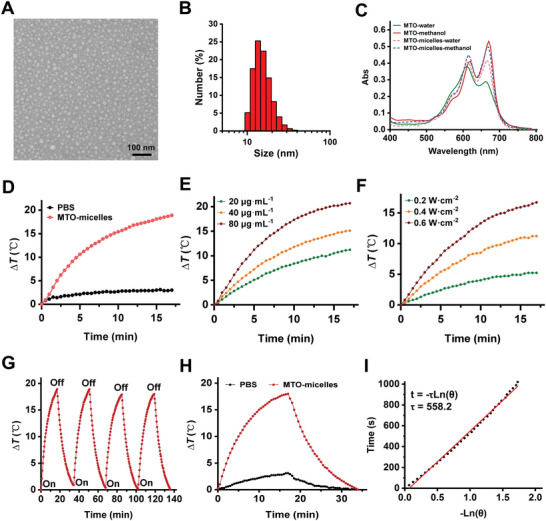
Characterizations of MTO‐micelles. A) The TEM image and B) DLS measurement of MTO‐micelles. Scale bar: 100 nm. C) UV–vis absorption spectra of free MTO and MTO‐micelles in aqueous and methanol solution (10 µg mL^−1^ of equivalent MTO concentration), respectively. D) Photothermal heating curves of PBS and MTO‐micelles (80 µg mL^−1^ of equivalent MTO concentration) under 665 nm laser irradiation (0.6 W cm^−2^). E) Photothermal heating curves of MTO‐micelles at various concentrations (20, 40, and 80 µg mL^−1^) under 665 nm laser irradiation (0.6 W cm^−2^). F) Photothermal heating curves of MTO‐micelles (60 µg mL^−1^) under 665‐nm irradiation under various laser intensities (0.2, 0.4, and 0.6 W cm^−2^). G) Photothermal stability of MTO‐micelles (100 µg mL^−1^ of equivalent MTO concentration) exposed to a 665‐nm laser (0.6 W cm^−2^). H) Photothermal heating/cooling curves of MTO‐micelles aqueous solution (100 µg mL^−1^ of equivalent MTO concentration, 1 mL) in the laser‐on and off state. Δ*T* stands for the temperature increasement of the solution. I) The time constant for MTO‐micelles heat transformation (*τ*) was calculated by plotting linear time data from the system cooling period against the negative natural logarithm of the system driving force temperature.

In this work, MTO‐micelles were prepared by our previous methods.^[^ ^32^ ^]^ Micellar shape and size were depicted in transmission electron microscopy (TEM) images and dynamic light scattering (DLS) data. According to **Figure** **1**A,B, MTO‐micelles was virtually spherical with an average size of 15–20 nm and a narrow size distribution. The micellar morphology and size did not change significantly after 30 d of storage at 4 °C in aqueous solution, demonstrating that MTO‐micelles maintained good stability. (Figures S1 and S2, Supporting Information). Similarly, the surface charge is also a criterion for micelle stability. The zeta potentials of MTO‐micelles remained fairly constant at around −14.5 mV before and after 30 d (Figure S3, Supporting Information), demonstrating that the micelles were stable in aqueous solutions.

The optical absorption of MTO and MTO‐micelles in different polarity solutions was analyzed using an UV–visible spectrometer. As shown in Figure 1C, free MTO dissolved in deionized water showed an absorbance spectrum with two maxima at 610 and 660 nm, corresponding to the dimer and monomer forms of the drug, respectively.^[^ ^33^ ^]^ These absorption peaks exhibited a bathochromic shift (redshift) when MTO was encapsulated in DSPE‐PEG micelles. The monomer absorption maxima shifted from 660 nm in water to 669 ± 1 nm when loaded within micelles, similar to that of MTO in methanol. The dimer peak showed a smaller redshift. Furthermore, at equivalent MTO concentrations, encapsulation resulted in an increase in the drug's overall absorption intensity profile. This is consistent with other findings on MTO absorbance in cetyltrimethylammonium bromide and sodium dodecyl sulfate micelles, and is likely since some drugs have lower absorption in aqueous media than in hydrophobic environments.^[^ ^34^ ^]^ These findings suggested that MTO was located at the hydrophobic core of DSPE. In addition, the standard curves showed an excellent linear correlation between MTO concentration and maximal absorbance at 669 nm in methanol solution (Figure S4, Supporting Information). And the encapsulation efficiency (EE) and drug loading (DL) of MTO‐micelles were calculated to be 93.07% and 11.58%, respectively. As a result, an MTO: DSPE‐PEG weight ratio of 1.5:10 is appropriate for preparing MTO‐micelles.

### 2.2 Photothermal Effect and Photothermal Conversion Efficiency of MTO‐Micelles

The photothermal property and photostability of MTO‐micelles are critical prerequisites for achieving satisfactory antitumor activity in vitro and in vivo.^[^ ^7^ ^]^ Given the significant NIR absorption of MTO‐micelles (Figure 1C), its photothermal properties in an aqueous solution were further explored. Figure 1D shows that after 17 min irradiation, the temperature of MTO‐micelles increased by 18.9 °C at a concentration of 80 µg mL^−1^ of equivalent MTO concentration, whereas the temperature of phosphate‐buffered saline (PBS) increased inconspicuously under the same 665 nm laser irradiation. There was no significant difference in temperature between MTO‐free and MTO‐micelles after 17 min irradiation (Figure S5, Supporting Information). It is conceivable to conclude that encapsulating MTO in the micellar hydrophobic core would not interfere in its photothermal performance. Under laser irradiation, the temperature of the solution gradually rose as the concentration of MTO‐micelles increased (Figure 1E). Besides, at the same concentration of MTO‐micelles, the temperature of the solutions increased steadily with the increase in laser power (Figure 1F). The temperature of MTO‐micelles could be stably increased from the base temperature to the maximum temperature over four repeated heating/cooling cycles, demonstrating that MTO‐micelles had good photothermal stability (Figure 1G). The temperature change processes of MTO‐micelles were recorded by turning the laser on/off, and the photothermal conversion efficiency of MTO‐micelles was calculated as 54.62% from the cooling process curve (Figure 1H,I). Together, these findings suggested that MTO‐micelles could serve as stable and efficient PTAs for precise PTT.

### 2.3 Cellular Uptake

**Figure 2:**
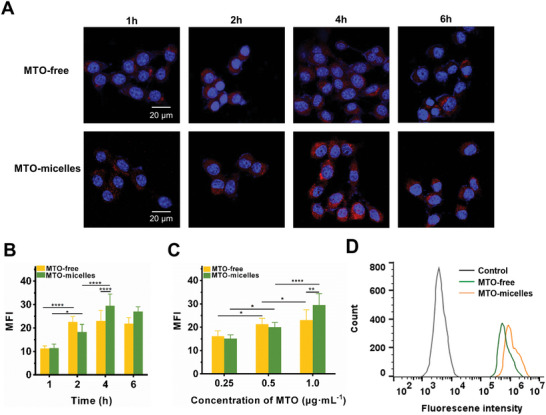
Cellular uptake of MTO‐micelles. A) Representative CLSM photographs and B) quantitative fluorescence analysis of 4T1 cells following incubation with free MTO or MTO‐micelles (1.0 µg mL^−1^ of MTO‐equivalent concentration) at various time intervals. The nucleus was stained with DAPI (blue; ex: 358 nm, em: 461 nm), and MTO moieties exhibited red channel fluorescence emission (ex: 633 nm, em: 680 nm). Data are presented as the mean ± SD (*n* = 3) (*P < 0.05 and ****P < 0.0001). C) Quantitative fluorescence analysis of 4T1 cells incubated for 4 h with various concentrations of MTO‐equivalent. Data are presented as the mean ± SD (*n* = 3) (*P < 0.05, **P < 0.01, and ****P < 0.0001). D) Flow cytometric measurement of 4T1 cells incubated for 4 h with free MTO or MTO‐micelles (1.0 µg mL^−1^ of MTO‐equivalent concentration).

The cellular membrane represents a physical barrier to mitoxantrone that the drug must penetrate to efficiently exert its biological activity.^[^ ^35^, ^36^ ^]^ Hence, the cellular uptake of MTO and MTO‐micelles was studied using confocal laser scanning microscopy (CLSM) and flow cytometry. According to **Figure** **2**A,B, the intracellular fluorescence intensity of the MTO‐micelles group maximized after 4 h of incubation. However, after 2 h of incubation, the fluorescence intensity of the MTO‐free group tended to be steady. The cellular uptake of MTO‐free and MTO‐micelles was concentration‐dependent under the same incubation duration (4 h). Furthermore, the cellular uptake of MTO‐micelles at a high concentration (1.0 µg mL^−1^) was significantly higher than that of MTO‐free (Figure 2C). The flow cytometry results further verified the excellent uptake of MTO‐micelles by 4T1 cells (Figure 2D). Because of their amphiphilic qualities, DSPE‐PEG polymers have been reported to be transported from micelles to the cell membrane and then ingested via non‐specific endocytosis.^[^ ^37^ ^]^ Consequently, hydrophobic drugs can be encapsulated in the inner core of micelles, and the micelles remain intact in the medium during cell incubation. When these micelles disassemble and release their payloads on the cell membrane, the released drugs accelerated into the cells and increased their concentration in the cells due to the increased membrane fluidity induced by PEG‐DSPE insertion. Therefore, MTO‐micelles could be exploited as a stable nanodrug delivery system.

### 2.4 In Vitro *γ*H2AX and HSP70 Expression

**Figure 3:**
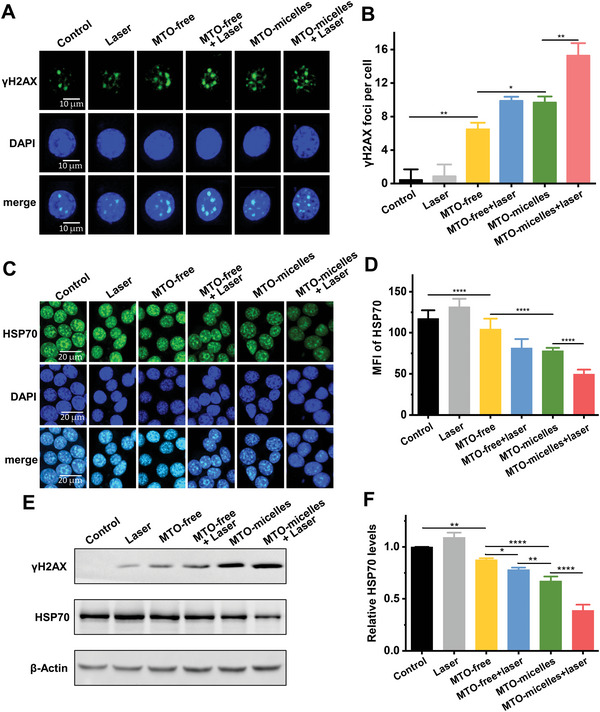
In vitro *γ*H2AX foci formation and HSP70 expression evaluation. A) Representative CLSM images and B) quantitative analysis of *γ*H2AX foci formation in 4T1 cells of different treatment groups. Data shown are mean ± SD (*n* = 20) (*P < 0.05, **P < 0.01). C) Representative CLSM images and D) quantitative analysis of HSP70 expression in 4T1 cells of different treatment groups. Data shown are mean ± SD (*n* = 10) (****P < 0.0001). E) Western blots of *γ*H2AX and HSP70 with *β*‐Actin as the internal reference, and F) Relative expression levels of *γ*H2AX and HSP70 in 4T1 cells of different treatment groups (MTO concentration was 1.0 µg mL^−1^, under 24 h of incubation). Data shown are mean ± SD (*n* = 3) (*P < 0.05, **P < 0.01, ****P < 0.0001).

Previous studies showed that PTT could induced chemotherapy sensitization via alleviate tumor hypoxia, and chemotherapeutic medicines could help reducing tumor cell thermotolerance, promoting PTT.^[^ ^38^, ^39^ ^]^ It is well known that cells can protect themselves against heat damage by upregulating HSPs.^[^ ^40^ ^]^ HSP70 is an inducible heat shock protein that is highly conserved. It belongs to the heat shock protein family and performs numerous physiological and pathological activities.^[^ ^41^ ^]^ Thus, immunofluorescence staining and Western blotting were used to determine the effect of chemotherapy and PTT on DNA damage and HSP70 expression. Mild hyperthermia (laser group) did not cause DNA damage on its own (**Figure** **3**A,B,E), with slight upregulation of HSP70 expression (Figure 3C–F). However, mild‐photothermal combined chemotherapy (MTO + laser) could increase DNA damage and decrease HSP70 expression. Furthermore, MTO‐micelles + laser induced the highest amount of *γ*H2AX foci and the least amount of HSP70 expression (Figure 3C–F). MTO has been shown to cause immunogenic cell death in tumor cells that could induce endoplasmic reticulum (ER) stress, resulting in protein release.^[^ ^42^, ^43^ ^]^ ELISA was used to determine the amount of HSP70 in tumor cell supernatants after administration. There was no significant difference in HSP70 levels between the groups (Figure S6, Supporting Information). Previous research has shown that chemotherapeutic agents that cause DNA damage can regulate the expression of a wide range of proteins, including the downregulation of HSPs.^[^ ^44^ ^]^ This consists to our findings. As a result, MTO could be utilized not only as a photothermal reagent with near‐infrared absorption, but also as a chemotherapeutic agent triggering DNA damage‐induced HSP70 expression reduction.

### 2.5 In Vitro Cytotoxicity

**Figure 4:**
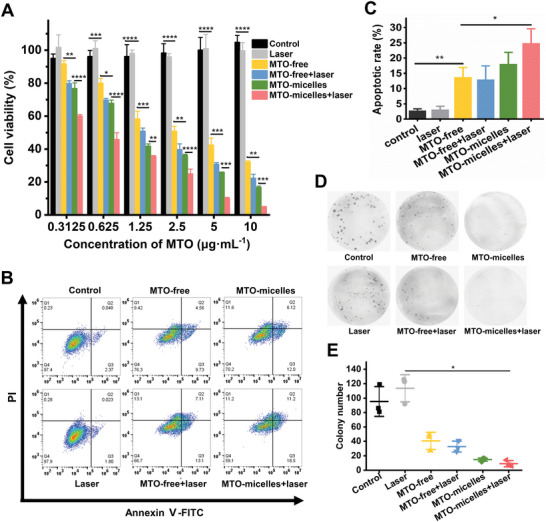
In vitro antitumor efficiency of MTO‐micelles. A) In vitro viability of 4T1 cells treated with laser (665 nm, 0.6 W cm^−2^, 2 min), MTO‐free, MTO‐free + laser (665 nm, 0.6 W cm^−2^, 2 min), MTO‐micelles, and MTO‐micelles + laser (665 nm, 0.6 W cm^−2^, 2 min) by CCK‐8 assay. Data shown are mean ± SD (*n* = 3) (*P < 0.05, ** *P* < 0.01, *** *P* < 0.001, and **** *P* < 0.0001). B) Flow cytometry analysis of 4T1 cells apoptosis induced by different treatments. MTO equivalent concentration was 1.0 µg mL^−1^. The incubation time was 24 h. Annexin‐positive cells were regarded as apoptotic cells. C) Apoptotic rate of different treatment groups determined by Annexin V‐FITC/PI staining. Data shown are mean ± SD (*n* = 3) (*P < 0.05, **P < 0.01). D) Representative images and E) quantitative analysis of the colonies of different treatment groups. Data shown are mean ± SD (*n* = 3) (*P < 0.05).

In consideration of the efficient cellular uptake of MTO‐micelles, we used CCK‐8 assay to further investigate the cytotoxicity of free MTO and MTO‐micelles with or without NIR irradiation. MTO‐micelles + laser had the highest cytotoxicity compared to the other treatments, including MTO‐micelles, MTO‐free + laser, or MTO‐free (**Figure** **4**A). The half maximal inhibitive concentration (IC50) of MTO‐micelles + laser was 0.55 µg mL^−1^, which was 2.25, 3, and 5.64 times lower than MTO‐micelles, MTO‐free + laser, and MTO‐free, respectively (Table S1, Supporting Information). The superior antitumor efficacy is attributed to the higher cellular internalization of MTO‐micelles and chemotherapy/PTT cotherapy as evidenced by HSP70 downregulation (Figure 3C–F).

Encouraged by the excellent cytotoxicity and synergistic therapeutic effect, we used flow cytometry to determine apoptosis of 4T1 cells treated with MTO‐free or MTO‐micelles with and without NIR irradiation. As demonstrated in Figure 4B,C, MTO‐micelles induced the highest apoptosis rate under NIR irradiation. Moreover, colony formation assays revealed that the photothermal thermotherapy of the MTO‐micelles could effectively inhibit cancer cell proliferation (Figure 4D,E). These findings were consistent with the CCK‐8 assay, which demonstrated that the combined mild‐photothermal chemotherapy had a significantly improved therapeutic efficacy.

### 2.6 Intratumoral Accumulation of MTO‐Micelles and Photothermal Effect

**Figure 5:**
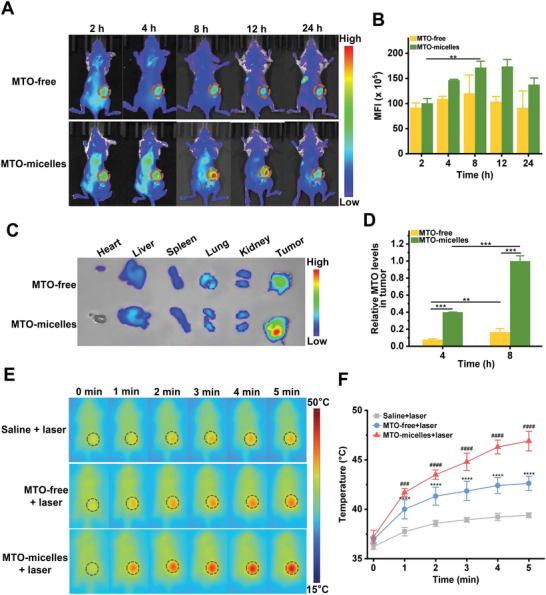
Intratumoral accumulation of MTO‐micelles and photothermal effect In vivo. A) Fluorescence images and B) mean fluorescence intensity of 4T1 tumor‐bearing mice at various time points following administration of MTO·2HCl or MTO‐micelles (5 mg kg^−1^ of equivalent MTO). Tumor sites are denoted by red dashed line circles. Values shown are mean ± SD (*n* = 3) (**P < 0.01). C) Ex vivo fluorescence images of major organs and tumors obtained 24 h after administration. D) HPLC analysis of MTO content in mice tumor tissues following intravenous administration of MTO·2HCl or MTO‐micelles (5 mg kg^−1^ of equivalent MTO). Values shown are mean ± SD (*n* = 3) (**P < 0.01, ***P < 0.001). E) Representative in vivo thermal images of 4T1 tumor‐bearing mice, and F) intratumoral temperature curves following intravenous injections of saline, MTO·2HCl, or MTO‐micelles (5 mg kg^−1^ of equivalent MTO) under NIR irradiation (665 nm, 0.6 W cm^−2^). Values shown are mean ± SD (*n* = 3) (****compared with saline + laser, *P* < 0.0001; #### compared with MTO‐free + laser, *P* < 0.0001).

Subsequently, we investigated the potential of MTO‐micelles for intratumoral accumulation and photothermal effect in 4T1 tumor‐bearing mice. To begin, real‐time fluorescence imaging was used to track the in vivo behaviors of MTO‐micelles post intravenous injection. Mice treated with MTO‐micelles showed high fluorescence signals at the tumor site, indicating efficient tumor accumulation of the MTO‐micelles. The maximal accumulation occurred around 8 h postinjection, and the fluorescence intensity could continue to be high for 24 h or even longer, most likely due to the EPR effect (**Figure** **5**A). On the contrary, the MTO‐free group showed lower fluorescence intensity than the MTO‐micelles group, indicating that free MTO was rapidly cleared from blood circulation (Figure 5B). Ex vivo fluorescence images of major organs (heart, liver, spleen, lung, and kidney) and tumors were obtained 24 h postinjection, further demonstrating the accumulation of MTO‐micelles in tumors (Figure 5C). In line with the findings of fluorescence imaging, high‐performance liquid chromatography (HPLC) analysis revealed that at 4 and 8 h after intravenous injection, intratumoral accumulation of MTO in the MTO‐micelles group was significantly higher than that in the MTO‐free group (Figure 5D and Figure S7, Supporting Information). These data strongly indicated that MTO‐micelles could be exploited as a stable nanodrug delivery system.

Photothermal imaging was employed to examine the in vivo photothermal conversion effectiveness of MTO‐micelles. NIR irradiation was implemented 8 h after intravenous injection of MTO‐micelles to maximize the therapeutic effects. As demonstrated in Figure 5E, the intratumoral temperature of mice injected with MTO‐micelles was significantly higher than that of the other groups at each time point under irradiation. After 5 min of irradiation, the average temperatures in the saline + laser and MTO‐free + laser groups barely rose by 3.2 and 5.7 °C, respectively, whereas the average temperature of tumors treated with MTO‐micelles rose rapidly from 37.2 to 46.9 °C, indicating a high potential of MTO‐micelles for inducing mild‐temperature (<50 °C) PTT effects for tumor therapy (Figure 5F). These results were attributed to the excellent in vivo stability and photothermal conversion properties of mto micelles, along with the EPR effect, which facilitated nanoparticle accumulation in tumor tissues. Encapsulating hydrophobic small molecule chemotherapeutic medicines in a micellar system can dramatically improve their pharmacokinetics and consequently their therapeutic impact.^[^ ^30^, ^32^ ^]^

### 2.7 In Vivo Antitumor Efficacy and Biocompatibility

**Figure 6:**
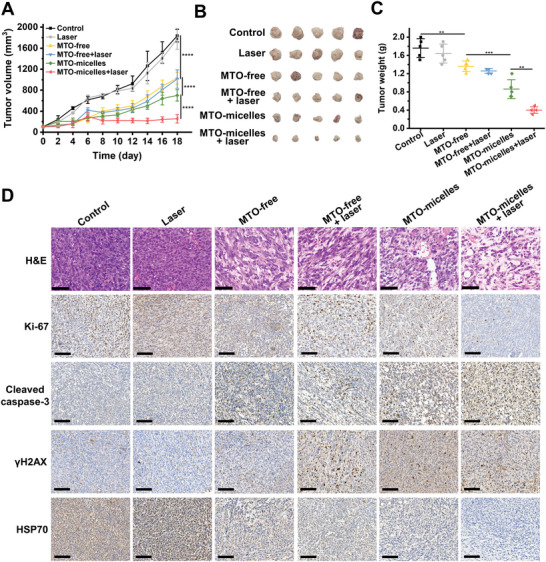
In vivo mild‐photothermal chemotherapy and histopathological analysis of tumor tissues. A) Tumor growth curves of mice during therapy. Values shown are mean ± SD (*n* = 5) (****P < 0.0001). B) The photographs of the extracted tumors at day 18 following various treatments. C) Average weights of tumors at day 18 following various treatments. Values shown are mean ± SD (*n* = 5) (****P < 0.0001). D) Representative H&E and IHC (Ki‐67, cleaved caspase‐3, *γ*H2AX, and HSP70) staining images of mice tumor tissues at the end of treatment. Scale bar: 100 µm.

Given the promising intratumoral accumulation and photothermal effect of MTO‐micelles, in vivo antitumor efficacy of mild‐photothermal chemotherapy was further investigated. The tumor growth curves of the therapy groups showed distinct differences (**Figure** **6**A). The mice treated with MTO (with or without laser) showed moderate inhibition of tumor growth in comparison to the control and laser groups. MTO‐micelles plus laser exhibited considerably stronger antitumor inhibitory effectiveness than any other groups, suggesting the considerable combination effects of mild‐PTT and chemotherapy. This was further confirmed by the photographs and weights of tumors exercised at the end of treatments (Figure 6B,C).

As the hematoxylin and eosin (H&E) and immunohistochemical (IHC) staining images shown in Figure 6D, tumor tissues in the MTO‐micelles + laser group exhibited significant remission area, lowest expression of Ki‐67, and greatest expression of cleaved caspase‐3, indicating that the NIR irradiation‐activated synergistic mild‐PTT and chemotherapy performed better in vivo than the single treatment. To explore the mechanism of MTO‐micelles‐mediated synergistic mild‐photothermal chemotherapy in vivo, tumors were extracted from mice 24 h after treatment and sectioned for IHC staining of *γ*H2AX and HSP70. Tumor tissues in the MTO‐micelles + laser group exhibited the highest *γ*H2AX expression and the lowest HSP70 expression (Figure 6D). The overall data showed herein provide insights into the activation of cell death processes induced by MTO‐micelles + laser, demonstrating the interplay between DNA damage and HSP70 expression.

MTO is an immunogenic cell death inducer that can activate TME and tumor‐specific cytotoxic T lymphocytes (CTLs) by initiating an ICD cascade.^[^ ^45^ ^]^ As a result, we performed ELISA assays to measure the expressions of HMGB1 and ATP in order to determine if MTO‐micelles combined with NIR can enhance the immunogenic cell death of tumor cells. MTO did promote immunogenic death of tumor cells, as demonstrated, but there was no significant difference in the expression of tumor cell immunogenic death markers between MTO‐free and MTO‐micelles (with or without laser) (Figure S8A,B, Supporting Information). Consistently, after 48 h of incubation with supernatant of 4T1 cells with various treatments, DC cell maturation showed no significant difference between MTO‐free, MTO‐free + laser, MTO‐micelles, and MTO‐micelles + laser groups (Figure S9A,B, Supporting Information). Furthermore, IHC staining revealed no CD8 expression in the tumor tissues of the control or laser groups. However, CD8 expression was found in the MTO‐free, MTO‐free + laser, MTO‐micelles, and MTO‐micelles + laser groups, but there was no significant difference between the groups (Figure S10, Supporting Information). In conclusion, MTO‐induced ICD contributed to the antitumor activity of the MTO nano‐system. However, given that there was no significant difference in ICD levels, DC cell activation and CTLs infiltration between the MTO‐micelles and MTO‐micelles + laser groups, MTO‐induced HSP70 inhibition may be the key explanation for the powerful antitumor efficacy in this case. Thus, the strong antitumor effect of MTO‐micelles combined with NIR is the synergistic results of NIR‐induced chemotherapy sensitization of MTO and downregulation of HSP70 expression caused by DNA damage of MTO.

To investigate the systemic toxicity in mice, we continuously monitored the change in body weight in all groups during the treatment period, confirming no significant body weight fluctuation (Figure S11, Supporting Information). There were no noticeable organ damage or inflammatory lesions observed in all major organs of mice receiving MTO‐micelles injection with or without photothermal treatment (Figure S12, Supporting Information). These data verified that MTO‐micelles rendered no obvious toxic side effects to the mice at the tested dosage.

## 3 Conclusion

In summary, MTO‐micelles have been effectively constructed for combined mild‐photothermal chemotherapy. MTO‐micelles have a nanosized (15–20 nm) structure with high drug loading and outstanding photothermal conversion efficiency. In vivo experiments revealed that MTO‐micelles could accumulate efficiently in tumor sites. Mild‐photothermal therapy after 665 nm NIR irradiation could successfully boost tumor cell susceptibility to chemotherapy. Moreover, MTO‐micelles as chemotherapeutic drugs could also downregulate the expression of HSP70, thereby reducing the heat endurance of tumor cells and enhancing the efficacy of mild‐photothermal therapy. Thus, the multifunctional MTO‐micelles, which could effectively eliminate tumors under mild‐hyperthermia, have a significant clinical utility.

## 4 Experimental Section

### Materials

DSPE‐PEG_2000_ was purchased from A. V. T. (Shanghai) Pharmaceutical Tech Co., Ltd. MTO and MTO dihydrochloride was obtained from Anhui Zesheng Technology Co., Ltd. Methanol was supplied by Aladdin Biochemical Technology Co., Ltd. 2‐(4‐Amidinophenyl)‐6‐indolecarbamidine dihydrochloride (DAPI), cell counting kit‐8 (CCK‐8), and Annexin V‐FITC/PI apoptosis detection kit were purchased from Beijing Solarbio Science & Technology Co., Ltd. RPMI‐1640 medium was provided by Vicente Biotechnology, Inc. Fetal bovine serum (FBS), penicillin, and streptomycin were purchased from Invitrogen. Phospho‐histone H2AX (Ser139) rabbit polyclonal antibody was purchased from Shanghai Beyotime Biotechnology Co., Ltd. Cleaved caspase‐3 (Asp175) antibody, HSP70 antibody, anti‐CD80‐FITC, and anti‐CD8 antibody was bought from Affinity Biologicals Inc. Mouse HSP70 ELISA kit, HMGB1 ELISA kit and ATP assay kit were bought from Shanghai Beyotime Biotechnology Co., Ltd. Murine 4T1 breast cancer cells and DC2.4 cells were cryopreserved in the lab. Female BALB/c mice (six weeks old, SPF class) were obtained from Beijing Vital River Laboratory Animal Technology Co., Ltd., and raised at the National Center for Nanoscience and Technology. All animal experiment operations were in accordance with the “Regulations on the Administration of Laboratory Animals” and guidelines from the National Center for Nanoscience Animal Health and Use Committee (NCNST22‐2207‐0716).

### Preparation and Characterization of MTO‐Micelles

MTO‐micelles were synthesized by the thin film‐hydration method.^[^ ^32^ ^]^ To obtain the micelles, MTO (1.5 mg) and DSPE‐PEG_2000_ (10 mg) were mixed in methanol. The mixture was then dried to a thin film using a vacuum rotary evaporator and hydrated in distilled water with continuous stirring at room temperature for 8 h. After 30 min ultrasonication in an ice bath, the micelles solution was then freeze‐dried and stored at −20 °C for future usage. The morphology of the prepared micelles was examined using TEM (Hitachi HT7700, Japan). The hydrodynamic diameters and zeta potentials of micelles were determined using DLS size and zeta system (Zetasizer Nano‐ZS 3600, Brookhaven, England). Ultraviolet–visible absorption spectrums of MTO‐micelles were recorded by UV–visible spectrophotometer (Techcomp UV2500, China). The amount of MTO in micelles was measured by UV absorption spectroscopy at 669 nm, and the encapsulation efficiency (EE) and drug loading (DL) of MTO‐micelles were calculated according to the formulas presented.^[^ ^46^ ^]^ In brief, the EE and DL were calculated using the following equations

(1) $$\mathrm{EE}\;\left(\%\right)=\frac{\mathrm{weight}\;\mathrm{of}\;\mathrm{the}\;\mathrm{drug}\;\mathrm{in}\;\mathrm{MTO}-\mathrm{micelles}\;}{\mathrm{initial}\;\mathrm{amount}\;\mathrm{of}\;\mathrm{drug}}\;\times \;100$$

(2) $$\mathrm{DL}\;\left(\%\right)=\frac{\mathrm{weight}\;\mathrm{of}\;\mathrm{the}\;\mathrm{drug}\;\mathrm{in}\;\mathrm{MTO}-\mathrm{micelles}\;}{\mathrm{weight}\;\mathrm{of}\;\mathrm{the}\;\mathrm{MTO}-\mathrm{micelles}}\;\times \;100$$

### Photothermal Conversion Evaluation

The temperature fluctuations of MTO and MTO‐micelles (80 µg mL^−1^ of equal MTO concentration) aqueous solutions (1 mL) were measured utilizing a laser (EOSHOW FC‐665, China) and an infrared thermal camera (FLUKE Ti4300, USA). Furthermore, various concentrations of MTO‐micelles (20, 40, and 80 µg mL^−1^) were prepared and irradiated with a 665 nm laser (0.6 W cm^−2^) for 17 min. MTO‐micelles (60 µg mL^−1^) were exposed to a 665‐nm laser with various power densities (0.2, 0.4, and 0.6 W cm^−2^) for 17 min. The temperature was recorded during the irradiation. For the photothermal stability test, MTO‐micelles were irradiated with a 665‐nm laser at 0.6 W cm^−2^ for 17 min before the laser was turned off, and the solution was naturally cooled down to the original temperature for another 17 min. Four heating/cooling cycles were monitored repeatedly, and the temperature of the solution was recorded every 30 s. The photothermal conversion efficiency (*ƞ*) was determined using a method described in the literature.^[^ ^47^ ^]^

(3) $$\eta =\frac{hs\;\left(T_{\mathrm{Max}}-T_{\mathrm{surr}}\right)-Q_{\mathrm{Dis}}}{I\left(1-10^{-A_{665\;\mathrm{nm}}}\right)}$$

*h* denotes the heat transfer coefficient, *s* represents the surface area of the container, *Q*_Dis_ expresses heat dissipated from the laser mediated by the solvent and container, *I* represents the laser power (0.6 W cm^−2^), and *A* is the absorbance at 665 nm.

(4) $$hs\;=\frac{mC_{\mathrm{water}}}{\tau_{s}}\;$$

*m* is the mass of the solution, *C* denotes the specific heat capacity of the solution [*C*_water_ = 4.2 J (g °C)^−1^], and *τ*_s_ represents the associated time constant.

(5) $$t\;=\;-\tau_{s}\ln\left(\theta \right)$$

*θ* shows a dimensionless parameter, known as the driving force temperature.

(6) $$\theta \;=\frac{T-T_{\mathrm{surr}}}{T_{\mathrm{Max}}-T_{\mathrm{surr}}}\;$$

*T*_max_ and *T*_Surr_ are the maximum steady state temperature and the environmental temperature, respectively.

### Cellular Uptake

4T1 cancer cells were cultured in RPMI‐1640 medium containing 10% FBS, 1% penicillin, and streptomycin at 37 °C in 5% CO_2_ environment. The cells (5000 per well) were seeded in confocal microscope plates with culture medium and cultured for 24 h at 37 °C in 5% CO_2_ environment. The original medium was then replaced with fresh medium containing MTO or MTO‐micelles at the same MTO concentration (0.25, 0.5, and 1.0 µg mL^−1^). After incubation for 1, 2, 4, and 6 h, the cells were washed three times with cold PBS. The nucleus was stained with DAPI (blue; ex: 358 nm, em: 461 nm, indicated by false blue color). A CLSM (Zeiss Z‐760, Germany) was used to capture the fluorescence images. MTO was excited with a 633‐nm laser and emission was detected in the far‐red region (maximum at 685 nm; indicated by false red color). The mean fluorescence intensity (MFI) of MTO was analyzed by ImageJ. In addition, cellular uptake of MTO or MTO‐micelles (1.0 µg mL^−1^ of equivalent MTO concentration) was detected by flow cytometry (Beckman CytoFLEX, Germany) (excitation wavelength of 635 nm and emission wavelength of 661 nm) after incubation with 4T1 cells for 4 h.

### In Vitro *γ*H2AX Formation and HSP70 Expression Evaluation by Immunofluorescence Staining and Western Blotting Assay

Immunofluorescence staining was performed to examine the formation of *γ*H2AX foci and expression of HSP70. 4T1 cells (5000 per well) were seeded in confocal microscope plates with culture medium and cultured for 24 h at 37 °C in 5% CO_2_ environment. The original medium was then replaced with fresh medium containing MTO or MTO‐micelles at the equivalent MTO concentration (1.0 µg mL^−1^). As controls, untreated cells in the medium were used. After 4 h of incubation, the laser group, MTO‐free + laser group and MTO‐micelles + laser group were subjected to 2 min of NIR irradiation (665 nm, 0.6 W cm^−2^). After further 20 h of incubation, the cells were incubated for 2 h at room temperature with anti‐*γ*H2AX or anti‐HSP70 antibodies. Then the cells were washed with PBS and incubated with a fluorophore‐conjugated secondary antibody. Finally, the cells were counterstained with DAPI for 5 min to visualize the nuclei. Fluorescence images were captured using a CLSM. The numbers of *γ*H2AX foci per cell were recorded, and the MFI of HSP70 was analyzed using ImageJ.

For Western blotting assay, 4T1 cells (1 × 10^6^ per well) were seeded in six‐well plates with culture medium and cultured for 24 h at 37 °C in 5% CO_2_ environment. The original medium was then replaced with fresh medium containing MTO or MTO‐micelles at the equivalent MTO concentration (1.0 µg mL^−1^). As controls, untreated cells in the medium were used. After 4 h of incubation, the laser group, MTO‐free + laser group, and MTO‐micelles + laser group were subjected to 2 min of NIR irradiation (665 nm, 0.6 W cm^−2^). After further 20 h of incubation, Western blotting analyses were performed according to the protocols for the routine with antibodies against *γ*H2AX or HSP70, and actin was employed as a protein loading control.

### ELISA Assay for HSP70 Release of 4T1 Cells

In addition, the release of HSP70 into extracellular medium was studied by ELISA. Following the treatment procedure above, the culture medium was harvested, and the HSP70 concentration was measured using a mouse HSP70 ELISA kit, according to the manufacturer's instructions.

### In Vitro Cytotoxicity Assay

4T1 cells were seeded at 3000 cells per well in 96‐well plates. Following a 24‐h preincubation period, cells were treated as follows. Cells without any treatments were used in the control group. Cells in the laser group were exposed to NIR irradiation (665 nm, 0.6 W cm^−2^) for 2 min. Cells in the MTO‐free group were treated with free MTO (0.3125, 0.625, 1.25, 2.5, 5, 10 µg mL^−1^) for 4 h. In the MTO‐free + laser group, cells were cultured with free MTO (0.3125, 0.625, 1.25, 2.5, 5, 10 µg mL^−1^) for 4 h before being exposed to NIR irradiation (665 nm, 0.6 W cm^−2^) for 2 min. Cells in the MTO‐micelles group were treated with MTO‐micelles (0.3125, 0.625, 1.25, 2.5, 5, 10 µg mL^−1^ of equivalent MTO concentration) for 4 h. Cells in the MTO‐micelles + laser group were cultured with MTO‐micelles (0.3125, 0.625, 1.25, 2.5, 5, 10 µg mL^−1^ of equivalent MTO concentration) for 4 h before exposing to NIR irradiation (665 nm, 0.6 W cm^−2^) for 2 min. After further 20 h of incubation, cell viability was determined for all groups by CCK‐8 test.

### Apoptosis Analysis by Flow Cytometry

The Annexin V‐FITC/PI Apoptosis Detection Kit was used to detect cell Apoptosis. 4T1 cells were seeded at 1 × 10^6^ cells per well in six‐well plates and cultured for 24 h at 37 °C in 5% CO_2_ condition. The original medium was then replaced with fresh medium (1 mL) containing MTO or MTO‐micelles at the equivalent MTO concentration (1.0 µg mL^−1^). Untreated cells in the medium were used as controls. After 4 h of incubation, the laser group, MTO‐free + laser group, and MTO‐micelles + laser group were exposed to NIR irradiation (665 nm, 0.6 W cm^−2^) for 2 min. After another 20 h of incubation, cells were collected and stained with Annexin V‐FITC/PI kit for FCM analysis.

### Colony Formation Test

Colony formation experiment was used to evaluate cell proliferation capacity. 4T1 cells were seeded at 500 cells per well in six‐well plates and cultured for 24 h at 37 °C in 5% CO_2_ condition. The original medium was then replaced with fresh medium (1 mL) containing MTO or MTO‐micelles at the equivalent MTO concentration (1.0 µg mL^−1^). Untreated cells in the medium were used as controls. After 4 h of incubation, the laser group, MTO‐free + laser group and MTO‐micelles + laser group were exposed to NIR irradiation (665 nm, 0.6 W cm^−2^) for 2 min. Then the medium was then replaced with complete medium. When colonies developed over the next 7 d, the cells were stained with GIMSA staining solution. The colonies in each well were counted.

### Tumor Model

Subcutaneous 4T1 tumors were generated by injection of 2 × 10^6^ cells in 100 µL PBS into the right posterior flank of mice. Beginning on the third day after the injection, the tumors that formed were monitored using a caliper. The tumor volume was calculated using the formula *V* = *L* × *W* ^2^/2, where *L* represents the longest dimension and *W* represents the shortest dimension of the tumor. The tumor volume reached ≈100 mm^3^ after about one week.

### In Vivo Multimodal Imaging

To monitor the in vivo biodistribution of MTO, an IVIS Spectrum system (PerkinElmer, USA) was employed. The subcutaneous 4T1 tumor model was established in female BALB/c mice before fluorescence imaging and photothermal conversion evaluation. When the tumor volumes reached about 300 mm^3^, the tumor‐bearing mice were randomized to one of two groups (*n* = 3) and individually injected with 100 µL of MTO dihydrochloride or MTO‐micelles aqueous solution with an equivalent MTO dosage (5 mg kg^−1^) via tail intravenous injection. In vivo fluorescence imaging was performed before and 2, 4, 8, 12, and 24 h postinjection. The mice were sacrificed 24 h postinjection to dissect the main organs (heart, liver, spleen, lung, kidney) and tumors for ex vivo fluorescence imaging. Fluorescence signal intensity was measured by Living Image 4.4 software (PerkinElmer, USA).

For photothermal imaging, tumor‐bearing mice were intravenously injected with 100 µL of free MTO or MTO‐micelles aqueous solution with the equivalent MTO dosage (5 mg kg^−1^). For the control group, mice were treated with the same volume of saline. After 8 h postinjection, the tumors were irradiated with a 665 nm laser at a power density of 0.6 W cm^−2^ for 5 min. The intratumoral temperature was measured by a FLUKE infrared thermal camera.

### HPLC for MTO Quantification in Tumor Tissues

The subcutaneous 4T1 tumor model was established in female BALB/c mice. When the tumor volumes reached about 1000 mm^3^, the tumor‐bearing mice were individually injected with 100 µL of MTO dihydrochloride or MTO‐micelles aqueous solution with an equivalent MTO dosage (5 mg kg^−1^) via tail intravenous injection. After 4 or 8 h of administration, the tumor tissues were sectioned and washed with saline. 500 mg of tumor tissues was mixed with 400 µL of 20% ascorbic acid aqueous solution for preparing tissue homogenate. Afterward, 200 µL of tissue homogenate was mixed with 200 µL of protein precipitant [acetonitrile: methanol = 10:90 (v:v), containing 0.5 mol L^−1^ HCl], and vortexed for 30 s. After 30 min at room temperature, the tissue homogenate was centrifuged (14 000 r min^−1^) for 15 min. 200 µL of supernatant was taken for chromatography. To determine the retention time of MTO, fresh calibration standards of 0.3125, 0.625, 1.25, 2.5, and 5 mg mL^−1^ were prepared. And the areas under the peak was calculated.

The HPLC (Shimadzu Prominence LC‐20A liquid chromatograph) separations were performed on an analytical Zorbax SB‐C18 column (150 mm × 4.6 mm, 5 µm, Agilent). The mobile phase was 75:25 (v:v) acetonitrile:water and running at a flow rate of 1.0 mL min^−1^. The detection wavelength was 658 nm. The column temperature was 30 °C. The injection volume was 20 µL.

### In Vivo Mild‐Photothermal Chemotherapy

4T1 tumor‐bearing BALB/c mice were randomized to one of six groups (*n* = 5): control, laser, MTO‐free, MTO‐free + laser, MTO‐micelles, and MTO‐micelles + laser. Individually, the mice in each group were injected via the tail vein with the corresponding materials at the equivalent MTO concentration (5 mg kg^−1^). Mice in both the control and laser groups were given the same volume of saline. At 8 h postinjection, the tumors of the mice in the laser group, MTO‐free + laser group, and MTO‐micelles + laser group were exposed to 5 min of NIR irradiation (665 nm, 0.6 W cm^−2^). The tumor volume and body weight of the mice were recorded every other day. The mice were sacrificed after 18 d of therapy to assess toxicity. Major organs including the heart, liver, spleen, lung, and kidney were harvested, fixed in 10% formalin, paraffinized, and sectioned. The tumor sections were then stained with H&E and imaged under a digital microscope (Aperio VERSA, Leica). H&E, proliferation (Ki‐67), and apoptosis (cleaved caspase‐3) staining were performed on the relevant tumors.

### In Vivo DNA Damage and HSP70 Expression Evaluation

At 24 h after treatment, mice from each group were executed and the tumors were collected, fixed in 10% formalin, paraffinized, and then sectioned for IHC staining to evaluate the tumor DNA damage (*γ*H2AX) and HSP70 expression via a typical IHC procedure.

### ELISA Assays of the ICD Effect

4T1 cells (1 × 10^6^ per well) were seeded in six‐well plates with culture medium and cultured for 24 h at 37 °C in 5% CO_2_ environment. The original medium was then replaced with fresh medium containing MTO or MTO‐micelles at the equivalent MTO concentration (1.0 µg mL^−1^). As controls, untreated cells in the medium were used. After 4 h of incubation, the laser group, MTO‐free + laser group, and MTO‐micelles + laser group were subjected to 2 min of NIR irradiation (665 nm, 0.6 W cm^−2^). After further 20 h of incubation, the culture medium was harvested, and the HMGB1 and ATP concentration was measured using a mouse HMGB1 and ATP ELISA kit, according to the manufacturer's instructions.

### Flow Cytometry Analysis of DC Maturation In Vitro

DC2.4 cells (1 × 10^6^ per well) were seeded in six‐well plates with culture medium and cultured for 24 h at 37 °C in 5% CO_2_ environment. The original medium was then replaced with the supernatants of 4T1 cell following the treatment procedure above. After 48 h of incubation, DC2.4 cells were stained with anti‐CD80‐FITC and then analyzed by FCM.

### In Vivo IHC Staining of CD8^+^ T Cells

At the end of treatment, mice from each group were executed and the tumors were collected, fixed in 10% formalin, paraffinized, and then sectioned for staining with anti‐CD8 antibody via a typical IHC procedure.

### Statistical Analysis

The relevant experimental data were presented as mean ± standard deviation (SD). The samples were statistically analyzed using the Student's *t*‐test and one‐way analysis of variance (ANOVA). *P < 0.05 (supposed to be of statistical significance), **P < 0.01, ***P < 0.001, ^####^ and **** stand for *P* < 0.0001. The Graphpad Prism 8.0.1 was used for data statistics.

## Conflict of Interest

The authors declare no conflict of interest.

## Author Contributions

Z.C., S.L., and F.L. contributed equally to this work. Z.C. and S.L. designed the study, completed the experiments, and wrote the manuscript. C.Q. and X.L. designed the study and discussed the results. G.Q., J.W., B.X., F.Z., and L.M. collected data. F.L., X.‐J.L., and Y.X. designed the study, discussed the results, and reviewed the manuscript.

## Supporting information

Supporting Information

## Data Availability

The data that support the findings of this study are available in the supplementary material of this article.
