# Supplementary material for: DNA Damage Inducer Mitoxantrone Amplifies Synergistic Mild‐Photothermal Chemotherapy for TNBC via Decreasing Heat Shock Protein 70 Expression
Source: Adv Sci (Weinh). 2023 Apr 17;10(16):2206707. doi: 10.1002/advs.202206707 (PMC10238214; doi:10.1002/advs.202206707)
Supplement: Supplementary file 1 — Supporting Information [file ADVS-10-2206707-s001.pdf]

ADVANCED SCIENCE
Open Access

Supporting Information

for *Adv. Sci.*, DOI 10.1002/advs.202206707

DNA Damage Inducer Mitoxantrone Amplifies Synergistic Mild-Photothermal Chemotherapy for TNBC via Decreasing Heat Shock Protein 70 Expression

Zuqin Chen, Sunfan Li, Fangzhou Li, Cheng Qin, Xianlei Li, Guangchao Qing, Jinjin Wang, Bozhang Xia, Fuxue Zhang, Liangliang Meng, Xing-Jie Liang* and Yueyong Xiao**

Supporting Information

DNA Damage Inducer Mitoxantrone Amplifies Synergistic Mild-photothermal Chemotherapy for TNBC via Decreasing Heat Shock Protein 70 Expression

Zuqin Chen, Sunfan Li, Fangzhou Li, Cheng Qin, Xianlei Li, Guangchao Qing, Jinjin Wang, Bozhang Xia, Fuxue Zhang, Liangliang Meng, Xing-Jie Liang*, Yueyong Xiao*Author(s), and Corresponding Author(s)**

Table S1. Summary of IC50 values for inhibition of proliferation of 4T1 cells

Group	IC50 [μg mL^{-1}]
MTO-free	3.10
MTO-free + laser	1.65
MTO-micelles	1.24
MTO-micelles + laser	0.55

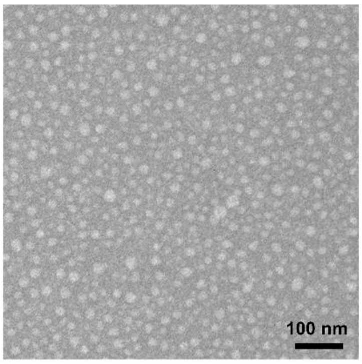

Figure S1. TEM image of MTO-micelles showed no significant change after 30 days storage at 4 °C. Scale bar: 100 nm.

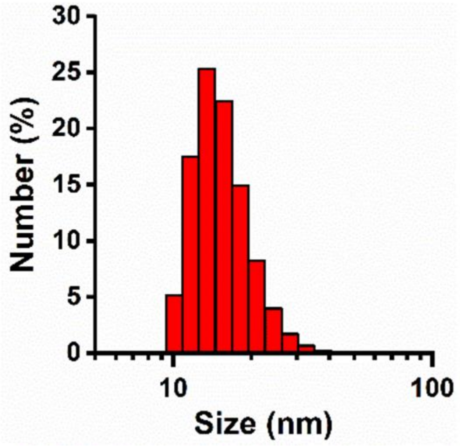

Figure S2. DLS measurement showed that the size of MTO-micelles had no significant change after 30 days storage at 4 °C.

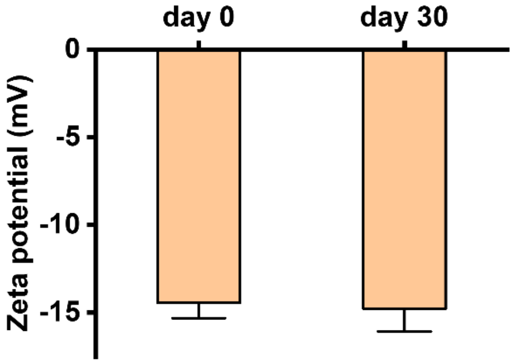

Figure S3. Comparison of zeta potential of MTO-micelles before and after 30 days storage at 4℃ (*n*= 3).

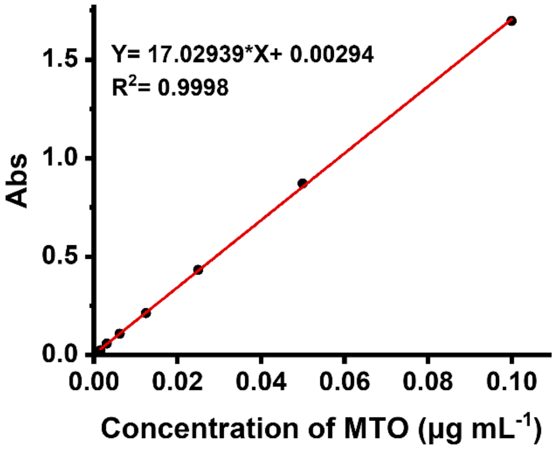

Figure S4. The concentration (μg mL^{-1}) of MTO as a linear regression function of the absorbance at a wavelength of 669 nm in methanol solution.

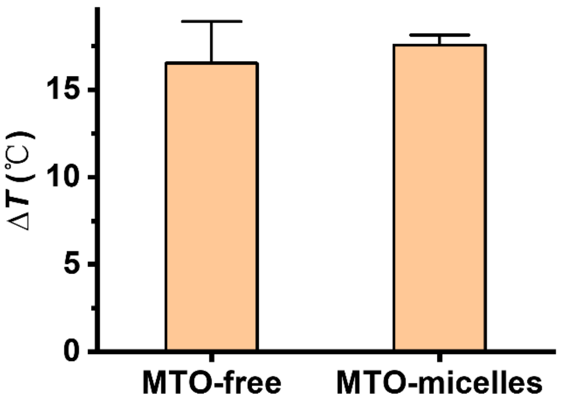

Figure S5. Comparison of the temperature increasement of MTO-free and MTO-micelles aqueous solution (80 μg mL^{-1} of equivalent MTO concentration) after 17 minutes irradiation (0.6 W cm^{-2}). ΔT represents the temperature increasement of solution (n= 3).

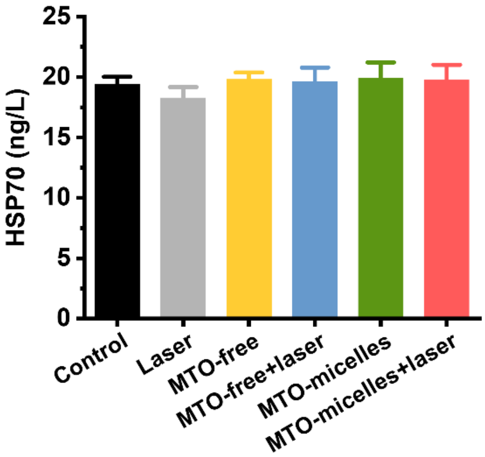

Figure S6. ELISA analysis of HSP70 release in the supernatants of tumor cells after different administration. Values shown are mean ± SD (*n*= 3).

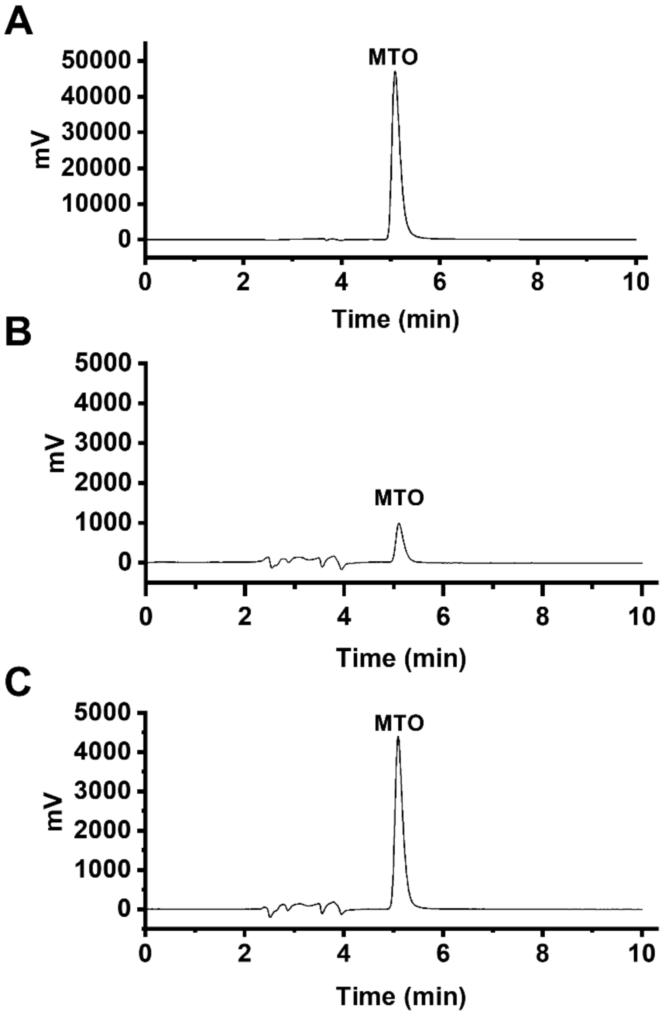

Figure S7. Representative HPLC chromatograms of A) standard control (MTO concentration was 5 μg mL^{-1}), B) MTO-free group and C) MTO-micelles group. MTO was eluted at ~5 minutes.

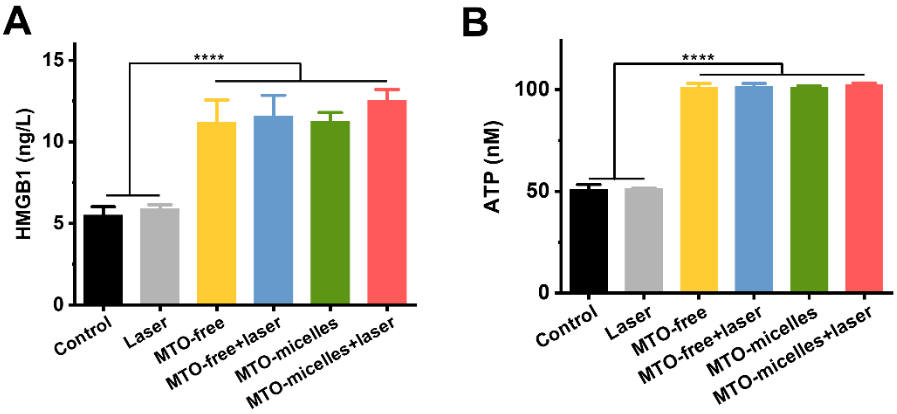

Figure S8. ELISA analysis of HMGB1 and ATP release in the supernatants of tumor cells after different administration. Values shown are mean ± SD (*n*= 3). (*****P* < 0.0001).

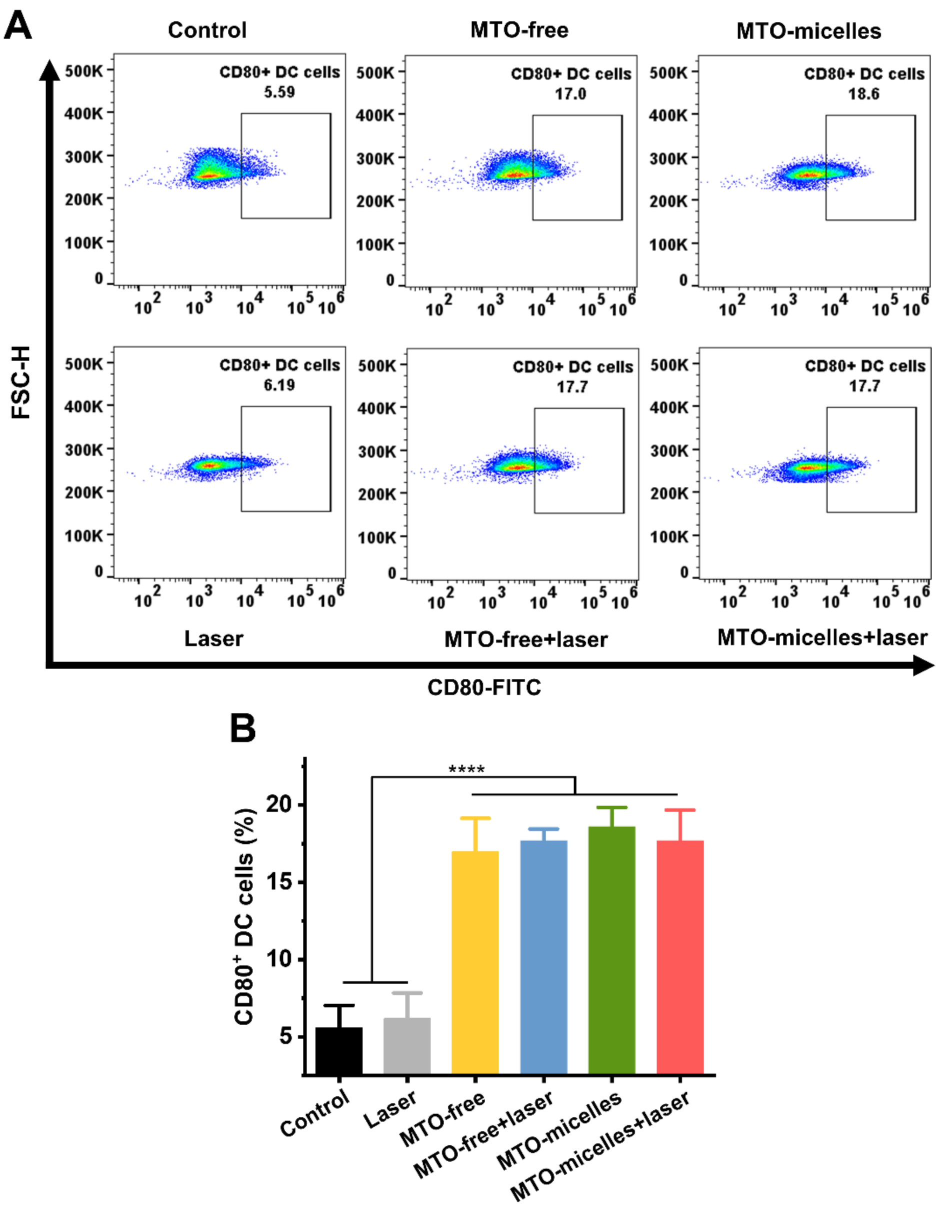

Figure S9. Maturation of dendritic cells. A) Representative FCM images and B) quantitative analysis of CD80 expression of DC2.4 cells after 48 hours of incubation with supernatant of 4T1 cells with different administrations. Values shown are mean ± SD (*n*= 3). (*****P* < 0.0001).

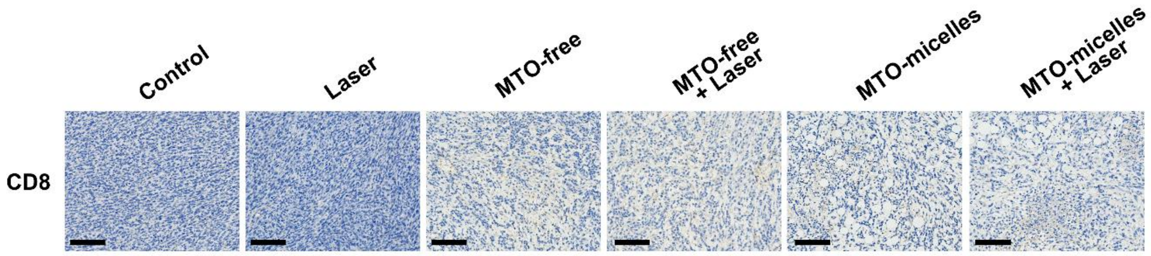

Figure S10. Representative IHC staining of CD8 images in tumor tissues at the end of treatment. Scale bar: 100 µm.

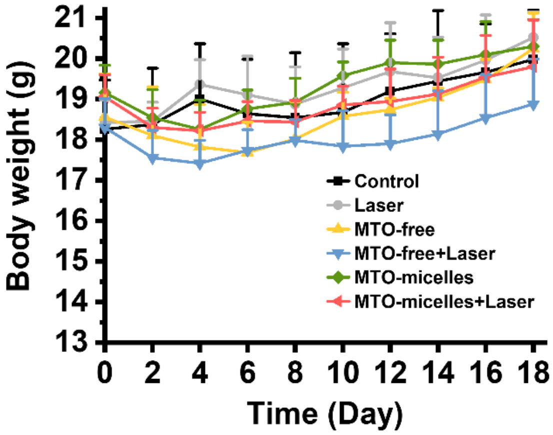

Figure S11. Curves of mice body weight throughout therapy. Values shown are mean ± SD (*n*= 5).

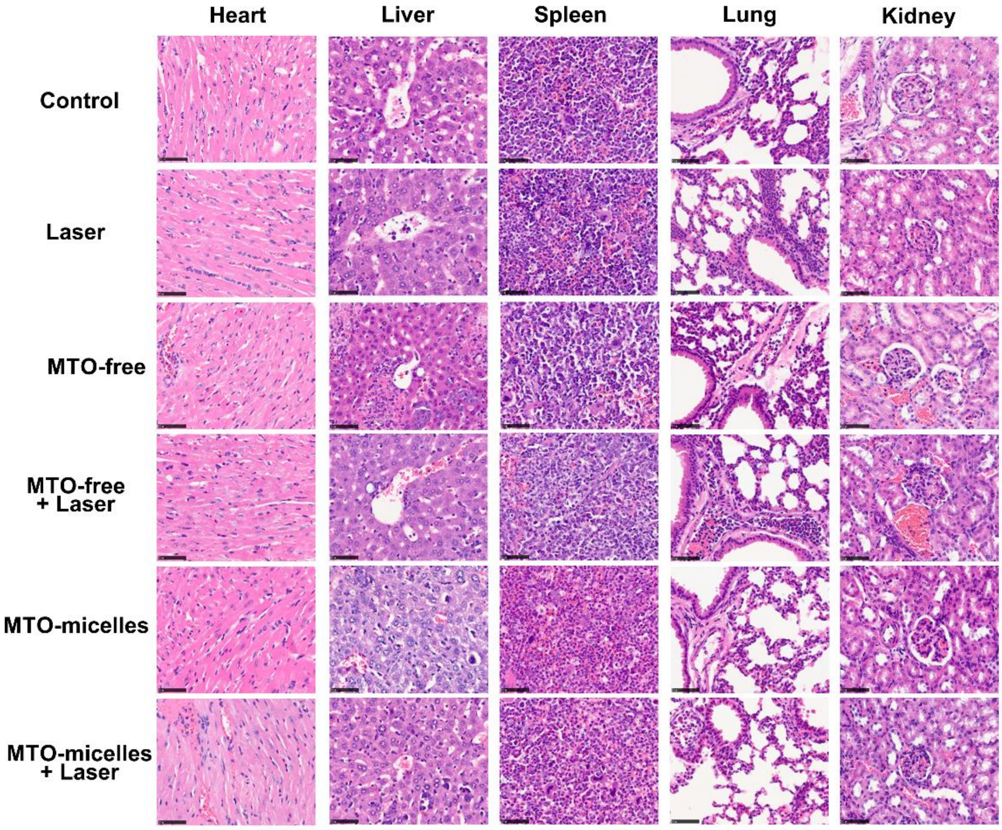

Figure S12. Representative H&E staining images of major organs (heart, liver, spleen, lung, and kidney) from mice at the end of treatment. Scale bar: 100 μm.
